# State Medical Examinations

**Published:** 1895-09

**Authors:** William Warren Potter

**Affiliations:** Buffalo, N. Y.; Member New York State Medical Examining and Licensing Board


### STATE MEDICAL EXAMINATIONS.

Conducted by WILLIAM WARREN POTTER, M. D., Buffalo, N. Y.
Member New York State Medical Examining and Licensing Board.

HEREWITH are appended statistics of the work of the New York State Boards of Medical Examiners for the academic year ending August 1, 1895, furnished from the regents' office and classified for the several examinations held during that period :

September, 1894, total number of candidates .................... 83

| | | Successful. | Rejected. | Per cent. Rejected. |
|---|---|---|---|---|
| State Board.... | 76 | 52 | 24 | 31.5 |
| Homeopathic... | 6 | 6 | .. | .... |
| Eclectic ....... | 1 | 1 | .. | .... |

November, 1894, total number of candidates .................... 77

| | | Successful. | Rejected. | Per cent. Rejected. |
|---|---|---|---|---|
| State.......... | 69 | 46 | 23 | 33.3 |
| Homeopathic .. | 5 | 4 | 1 | 20. |
| Eclectic ....... | 3 | 3 | .. | .... |

January, 1895, total number of candidates .................... 56

| | | Successful. | Rejected. | Per cent. Rejected. |
|---|---|---|---|---|
| State ......... | 49 | 33 | 16 | 32.6 |
| Homeopathic... | 6 | 6 | .. | .... |
| Eclectic ....... | 1 | 1 | .. | .... |

April, 1895, total number of candidates .................... 66

| | | Successful. | Rejected. | Per cent. Rejected. |
|---|---|---|---|---|
| State .......... | 61 | 44 | 17 | 27.8 |
| Homeopathic... | 5 | 5 | .. | .... |
| Eclectic ....... | .. | .. | .. | .... |

May, 1895, total number of candidates .................... 163

| | | Successful. | Rejected. | Per cent. Rejected. |
|---|---|---|---|---|
| State.......... | 138 | 96 | 42 | 30.4 |
| Homeopathic... | 24 | 21 | 3 | 12.5 |
| Eclectic. ...... | 1 | 1 | .. | .... |

June, 1895, total number of candidates .................... 232

| | | Successful. | Rejected. | Per cent. Rejected. |
|---|---|---|---|---|
| State.......... | 213 | 174 | 39 | 18.3 |
| Homeopathic... | 14 | 10 | 4 | 28.5 |
| Eclectic. ...... | 5 | 4 | 1 | 20. |

GRAND TOTALS.

| | | Successful. | Rejected. | Per cent. Rejected. |
|---|---|---|---|---|
| Total No. examined during year.......... | 677 | 507 | 170 | 25.1 |
| " " " by State Board....... | 606 | 445 | 161 | 26.5 |
| " " " " Homeopathic Board. | 60 | 52 | 8 | 13.3 |
| " " " " Eclectic Board..... | 11 | 10 | 1 | 9.1 |

### MEDICAL SYLLABUS.

The regents of the university have lately issued examination bulletin number seven, a medical syllabus, prepared under the direction of the state board of medical examiners. The purpose of this syllabus is to specify, for the guidance of candidates, the topics covered by the examinations that are now required to be taken under the statute by all applicants for license to practise medicine and surgery in this state. Candidates, however, need not flatter themselves that the examiners will take up these topics *seriatim* and propound questions based upon each head from beginning to end until all are exhausted. This would be a public advertisement of the methods pursued in the examinations and would, of course, thwart its purpose. Topics, however, will be chosen from this syllabus for a basis of each group of questions propounded ; nor will the examiners go outside of this syllabus in formulating the examination queries.

This syllabus is subject to revision from time to time, hence the latest edition should always be consulted. The bulletin can be obtained on application to the regents' office, Albany, and a remittance of 25 cents.

---

### THE ILLINOIS BOARD OF HEALTH.

The following preamble and resolution explains the issue between the Woman's Medical College and the Illinois board of health :

WHEREAS, The faculty of the Northwestern university woman's medical college adopted a set of resolutions, criticising the Illinois state board of health for having issued to three non-graduate students of said college the state certificate entitling them to practise medicine, whom they claim were not entitled to receive them, and charging the board with having adopted a lax policy in numerous other instances, thereby seriously detracting from the usefulness of the board ; and

WHEREAS, Although the resolutions were "ordered to be placed before the Illinois state board of health," they were furnished to the various medical publications of the country simultaneously with their presentation to the board and before the board had an opportunity to make any defense ; and

WHEREAS, The said college has not made any investigation of the methods or policy of the board, and could not be in possession of information upon which to found such serious charges ; and

WHEREAS, The secretary of the faculty admitted to the secretary of the board that the resolutions were adopted without due consideration and were not applicable to the present board; and

WHEREAS, In the past two years no certificate has been granted to any applicant upon an average rating of less than 80 per cent. on all branches, and the questions and examination papers and a tabulated record of all examinations are preserved, and are matters of record in the office; and

WHEREAS, It is not in the province of the board to adopt any policy regarding the admission to its examinations of non-graduates, the law prescribing that "non-graduates shall submit themselves for examinations," and further prescribing that "the examinations shall be of an elementary and practical character;" therefore, be it

*Resolved*, That justice demands that the faculty of the Northwestern university woman's medical college, and all others interested, inform themselves as to the methods and policy of the Illinois state board of health in conducting its examinations, with a view to the establishment of the charges made, or of making such withdrawal, alteration or explanation of the charges as the facts may warrant; and further, that the faculty inquire as to whether any individual interest or personal animosity prompted the drafting and circulating of the resolutions.

(Signed) B. M. GRIFFITH, M. D.,

SARAH HACKETT STEVENSON, M. D.

---

### MAINE MEDICAL REGISTRATION BOARD.

It affords the *Journal* much satisfaction to be able to give the minutes of the organisation of the board of registration at Augusta, to give the form of application for registration of medicine and to be able to state that registration of physicians of the state is going on as fast as could be expected.

The importance of the consummation of this work is appreciated only by a few, because but little attention has been given to it.

Its far-reaching effect can be estimated by reviewing the results of such a law in other states, like that of Minnesota.

To assist in the accomplishment of this grand result has been one of the highest aims of the *Journal*.

It will be remembered that Dr. Millard in his excellent paper, printed in our last issue, stated that "in a period of twelve years the proportion of physicians to the population in Minnesota has been reduced from one practitioner to every 650 in 1883, to one to every 1,000 in 1895." The state is substantially rid of the traveling charlatan.

*The London Lancet* in a recent editorial says :

It is a disagreeable reflection but a necessary and salutary one that the honor, dignity and general well-being of medical men depend upon the numbers of medical practitioners being kept within reasonable limits. If these limits be exceeded—and we take it that in this country and in America they have already been largely exceeded—the inevitable law of supply and demand comes into force ; the temptation to undersell their brethren becomes to many irresistible and the whole profession suffers in dignity.

Regulating the practice of medicine by efficient registration will not only diminish the number of physicians but it will increase their efficiency—two important things to uphold the honor and dignity of the profession.

Those who are eligible to registration should send to Dr. A. K. P. Meserve, 109 Emery street, Portland, for blanks, fill them out as indicated and send them in at once, to facilitate the work as much as possible.—*Journal of Medicine and Science.*

---

MEDICAL EXAMINERS FOR THE STATE OF GEORGIA.

Under the above title the *Southern Medical Record* publishes an editorial, written by Dr. J. McFadden Gaston, which gives a synopsis of the new law now in force in the state of Georgia. This article so admirably sets forth the new medical status of the empire state of the South that we quote it in full :

The bill which passed the legislature of Georgia for the regulation of the practice of medicine in the state, permits all who were entitled at the date it went into effect to exercise the office of physician or surgeon to continue in the discharge of their duties.

The large brood of quacks developed in the city of Atlanta, by the laxity of our laws heretofore, will doubtless presume upon this recognition to assert their claims to a place in the medical profession, from which they have been debarred by ethical standards. It, therefore, devolves upon the practitioners of good standing in the regular school to draw the lines now more distinctly than ever to protect the medical profession from the encroachments of those not conforming to the rules and regulations of the ethical code.

This applies not only to those holding diplomas from schools not recognised as regular, but to the graduates of regular schools who are not following the lines of practice inculcated by their schools, and by the Medical Association of Georgia under the code of ethics laid down for the guidance of the profession by the American Medical Association. There are now in this city and in other cities of Georgia, a large number of regular graduates who are engaged in the practice of quackery in its various forms, and so far as the enactment of the bill goes, they are at liberty to keep up those practices. But they are not entitled to recognition by the regular practitioner in pursuing this devious course and should be ruled out of the medical profession. Those who are openly resorting to a species of advertisement not consistent with medical ethics, should be placed by all medical men of good repute in the category of quacks, who are debarred from recognition by consultation or otherwise. If these members of the medical profession prefer such irregular procedures to a compliance with the code of ethics, we can only say that "Ephraim is joined to his idols, let him alone." But those who prize their good name in the profession should wipe their hands from the stain of professional association with all such degenerate sons of Æsculapius.

The working of the new law will effectually rule out all such vultures as Flower, who has been preying upon the people of Atlanta and other Southern cities during the past few years.

The traveling firms of so-called doctors, who have spent some weeks or months of each year in our city, have imposed upon the credulity of those suffering from imaginary ailments and have reaped a large harvest of pecuniary gain from people who were little able to meet their exactions and who received, in most cases, no benefit whatever from their treatment.

The authorities should keep a close watch upon these harpies in future and compel them to move on when disposed to stop here.

In respect to the bearing of the bill upon the medical colleges and the graduates of the same for the future, it remains to be seen what will be accomplished for the welfare of the community in supplying competent physicians.

The requirement of a three years' graded course of lectures of six months each, must necessarily afford better preparation for the practice of medicine and surgery than the former two years' course of five months each, with a simple repetition of the same lectures.

What is still of vital importance remains undone by this bill, and is not sufficiently insisted upon by any of the medical schools, that is, the preliminary training of the student by a competent preceptor before entering upon a course of medical lectures. Fifty years ago this requirement was fulfilled in the case of most students before going to a medical college, and when a young man went into the lecture-room with this preparation, he could take in more satisfactorily what was taught by the professors in the various departments.

This omission is not met by the requirement of the Southern Medical College Association for preparatory scholarship in the ordinary branches of knowledge before entering upon the study of medicine. However necessary this may be to properly comprehend the instruction of the lecture-room, it cannot supply the deficiency of a special preceptor in the rudimentary departments of anatomy, physiology, materia medica and chemistry, for the medical student.

Unfortunately, a large proportion of the young men who have entered our medical colleges in recent years, have not possessed the ordinary schooling or the elementary medical training which should prepare them for a course of lectures in a medical college.

The natural consequence of this lack of preparatory instruction is manifested by their inability to comprehend what is taught by the professors, and with the multiplicity of matters presented each day, they become confused and receive little profit.

This precipitation will be very materially modified by the operation of the medical examining board, which must tend to impress upon prospective students the importance of proper preparatory study.

The division of the medical examiners into three boards of five members each, which meets the diversity of teaching in the regular, eclectic and homeopathic school, ought to be satisfactory to all parties. If the men who have been appointed by the Governor to constitute the regular board of examiners do their duty faithfully and discreetly; no graduate of any regular school of medicine in Georgia who is fitted to enter upon the duties of his profession ought to be rejected.

If from any cause a young man gets hold of a diploma without proper qualification, this examination by the board is intended to put a check upon his wild career of adventure and by reminding him that he needs further instruction, a great benefit will be conferred upon him, and a greater still upon the community in which he expects to practise.

It is to be expected that personal influence may seek to gain favor for candidates at times, and yet the members of the board should prove themselves independent of all such favoritism.

The wise provision that no member of the medical board shall be connected with any of the medical colleges should prove an effectual guarantee against any partiality for the students of either school.

The competition of the different medical schools in Georgia will in future resolve itself into a decision in favor of that one which affords the best training for the duties of the physician and surgeon. A student will no longer raise the question as to the school in which he can get a diploma easiest and with the least outlay. His inquiry will be directed to getting assurance of the attainment of that knowledge of the different branches which will enable him to pass a satisfactory examination by the faculty for graduation and subsequently to meet the requirements of the board of medical examiners for the state of Georgia, who are sworn to do their duty fairly and justly. So mote it be. J. McF. G.

The following gentlemen are the appointees constituting the medical examining boards:

*Regular Board.*—Dr. F. M. Ridley, LaGrange, three years;

Dr. J. B. Baird, Atlanta, one year ; Dr. A. A. Smith, Hawkinsville, two years ; Dr. E. R. Anthony, Griffin, two years ; Dr. W. O'Daniel, Bullards, one year.

*Homoepathic Board.*—Dr. C. C. Schley, Savannah, three years; Dr. R. A. Hicks, Rome, one year ; Dr. M. A. Cleckley, Augusta, two years ; Dr. C. A. Geiger, Roswell, two years; Dr. E. B. Schley, Columbus, one year.

*Eclectic Board.*—Dr. M. T. Salter, Atlanta, one year ; Dr. M. K. Phillips, Bremen, two years ; Dr. J. F. Harris, Dalton, two years; Dr. J. Frank Harris, Thomas county, three years ; Dr. W. V. Robertson, Rehoboth, Morgan county, one year.

---

### RECENT MEDICAL LEGISLATION IN MINNESOTA.

That Minnesota is in the van of progress as regards keeping up a high standard of attainments among those allowed to practise medicine within the state, has been acknowledged ever since the passage of the first medical practice act ten years ago. By that act applicants for a license must have attended at least three courses of lectures of six months each. The legislature just adjourned has gone a step further and has passed a bill providing that of all graduates of a later date than 1898 must have attended at least "four courses of medical lectures, in different years, of not less than six months' duration." From this it is evident that no student who after this year enters a medical school with a three years' course will be admitted to practise medicine in Minnesota, unless he does enough post-graduate study to comply with the requirements.

The legislature was also liberal to the medical department of the State University and gave it a grant of $40,000 for the purpose of building a laboratory. This makes a total of $150,000 appropriated for buildings alone for this department during the last four years.—*Northwestern Lancet.*

---

### THE FOUR-YEAR COURSE.

Nothing which has occurred during the past ten years more definitely indicated the advancement of the science of medicine than the adoption of the four-year course. It has not been the result of any individual movement, but it has come from necessity. There is more to teach and consequently more time is required in which to treat it. The profession, the student and the public demand a more thorough preparation of the man who is to deal with human life. There should be no hesitancy on the part of colleges in making the full requirement. The number of students may be diminished, but the quality of the graduates will be so vastly improved that the credit of the institution must be somewhat enhanced. A student who refuses to come up to the highest standard of requirement, can be but little credit to the college which graduates him, or to the profession which he enters. In the competition for students it is a question if the short-time schools will not reap the greater harvest. But we believe the influence of the profession (and usually the student follows the advice of his preceptor) will be in favor of the four-year schools.—*Kansas Medical Journal.*

---

WASHINGTON STATE BOARD OF MEDICAL EXAMINERS.

The governor of Washington has appointed the following physicians to fill the vacancies on the state board of medical examiners of that state—namely, Dr. C. A. Smith, of Seattle; Dr. Henry W. Dewey, of Tacoma, and Dr. E. P. Penfield, of Spokane.—*Medical Bulletin.*

